# Neuropilin-2 Signaling Modulates Mossy Fiber Sprouting by Regulating Axon Collateral Formation Through CRMP2 in a Rat Model of Epilepsy

**DOI:** 10.1007/s12035-022-02995-0

**Published:** 2022-08-31

**Authors:** Yuxiang Li, Fangchao Tong, Yiying Zhang, Yiying Cai, Jing Ding, Qiang Wang, Xin Wang

**Affiliations:** 1 Department of Neurology, Zhongshan Hospital, Fudan University, Shanghai, China; 2 Department of The State Key Laboratory of Medical Neurobiology, MOE Frontiers Center for Brain Science, Institutes of Brain Science, Fudan University, Shanghai, China

**Keywords:** Neuropilin-2, Epilepsy, Mossy fiber sprouting, CRMP2, Axon collateral formation, Adult

## Abstract

Programmed neural circuit formation constitutes the foundation for normal brain functions. Axon guidance cues play crucial roles in neural circuit establishment during development. Whether or how they contribute to maintaining the stability of networks in mature brains is seldom studied. Upon injury, neural rewiring could happen in adulthood, of which mossy fiber sprouting (MFS) is a canonical example. Here, we uncovered a novel role of axon guidance molecule family Sema3F/Npn-2 signaling in MFS and epileptogenesis in a rat model of epilepsy. Dentate gyrus-specific Npn-2 knockdown increased seizure activity in epileptic animals along with increased MFS. Hippocampal culture results suggested that Npn-2 signaling modulates MFS via regulating axon outgrowth and collateral formation. In addition, we discovered that Sema3F/Npn-2 signal through CRMP2 by regulating its phosphorylation in the process of MFS. Our work illustrated that Npn-2 signaling in adult epilepsy animals could potentially modulate seizure activity by controlling MFS. MFS constitutes the structural basis for abnormal electric discharge of neurons and recurrent seizures. Therapies targeting Npn-2 signaling could potentially have disease-modifying anti-epileptogenesis effects in epilepsy treatment.

**Supplementary Information:**

The online version contains supplementary material available at 10.1007/s12035-022-02995-0.

## Introduction

Neuroplasticity is a defining feature of the nervous system. Programmed neural circuit formation during development lays the foundation for normal brain functions. However, under certain circumstances, neural rewiring occurs in adulthood, for example, mossy fiber sprouting (MFS) in epilepsy. Mossy fibers, the axon of granule cells, extend to mossy cells and inhibitory interneurons in the hilus before directionally projecting to and forming synapses with CA3 pyramidal neurons under normal conditions [1]. MFS was described in mesial temporal lobe epilepsy (mTLE) patients [2] and animal models of epilepsy [3]. It refers to the reversed and extensive innervation of mossy fibers to the inner molecular layer (IML) partly due to the vacancy of synaptic sites in hilus [4]. Sprouted mossy fibers mainly form synaptic contact with dendrites and dendritic spines of granule cells [5, 6], creating a local excitatory feedback circuit with the potential to synchronize neuronal firing. These excitatory neural loops form the structural basis for abnormal discharge of neurons and recurrent seizures.

Though the molecular mechanisms have not been fully understood, the 3-step axon guidance model helps to explain the process of MFS [7]. First, triggered by neuron loss in the hilus, robust mossy fiber collaterals branch to initiate the sprouting. Second, under the regulation of attractive and repulsive axon guidance molecules, sprouted mossy fibers reversely travel to enter and tightly confine in the IML. Third, the following mossy fibers fasciculate with the pioneer ones, which are guided by contact-dependent axon guidance cues. Axon guidance molecules play important roles in the process of MFS. MFS involves critical steps for neural network formation, including axon collateral formation and elongation, directional projection to target cells, and functional synapse formation, which makes it an ideal model to study neuronal circuit reorganization and plasticity in the mature hippocampus.

Neuropilin-2 (Npn-2), forming a co-receptor complex with plexinA3, mediates semaphorin 3F (Sema3F) in regulating axon guidance [8], axon pruning [9], dendritic spine remolding [10, 11], and other neuroplasticity modulations [12] during nervous system development. Sema3F or Npn-2 knockout mice showed increased spontaneous recurrent seizure (SRS) sensitivity, even handling-induced seizures [11, 13], possibly due to the reduction of GABAergic interneurons [11, 13, 14]. In addition to their functions in developmental stages, Npn-2 and Sema3F persiste their presence in adulthood and change significantly in adult TLE patients [15] and epileptic animal models [16, 17]. Therefore, Npn-2 signaling in adult animals is likely to involve in epilepsy. Given its crucial function in axon guidance and pruning, we think Npn-2 signaling is probable to modulate neural network remodeling in adulthood.

In this study, we used the hippocampus dentate gyrus-specific Npn-2 knockdown approach in adult animals to study its functions in mature brains. Pilocarpine-induced rat model of epilepsy was established to study the effects of adult-specific Npn-2 knockdown on MFS and epileptogenesis. Furthermore, we investigated molecular mechanisms of Npn-2 signaling in the process of MFS. Collapsin response mediator protein 2 (CRMP2) was found to be a promising candidate to mediate Npn-2 signaling in this process. Finally, we explored the cellular mechanisms of Npn-2 to regulate MFS.

## Materials and Methods

### Animals

Male adult Sprague–Dawley rats (200–250 g) were housed under a 12-h day-night cycle. The experiment was done in accordance with the guidelines of the National Institutes of Health. This study was approved by the Committee of Animal Care and Use in Zhongshan Hospital of Fudan University (Shanghai, China).

### Pilocarpine-Induced Rat Model of Epilepsy

Pilocarpine-induced rat model of epilepsy was established as described previously [18]. LiCl (127 mg/kg, Sigma-Aldrich, USA) was intraperitoneally (IP) injected 24 h before pilocarpine administration. Scopolamine methyl bromide (1 mg/kg, TCI, Japan) was given IP to reduce peripheral muscarinic effects. Thirty minutes later, pilocarpine (40 mg/kg, Sigma-Aldrich, USA) was IP injected to induce status epilepticus (SE). Seizure activity was evaluated with a modified Racine scale [19, 20]. Diazepam (10 mg/kg, King York, China) was intraperitoneally injected 60 min after SE onset. Rats were then monitored with a video surveillance system (JVC, Japan) for 21 days in transparent cages. The video from 08:00 to 20:00 per day was double-blindly analyzed.

### Intrahippocampal Injection

Adeno-associated viruses (AAV) used in this study were constructed by Genomeditech (China) and lentiviruses were constructed by Genechem (China). Adult rats were anesthetized with 1% pentobarbital sodium (5 mL/kg, IP) and fixed in a stereotaxic frame. One burr hole was drilled into each side of the skull using Bregma as the reference point (AP − 3.72 mm, ML ± 2.2 mm, DV − 3.4 mm). Two microliters of the virus was stereotaxically injected at each site at a rate of 0.2 μL per min. The syringe was retained for 5 min after injection to prevent intracranial hemorrhage.

### Real-Time Quantitative PCR

Total RNA was extracted with Tissue RNA Purification Kit (EZBioscience, USA). Five hundred nanograms of RNA of each sample was reverse transcribed with PrimeScript™ RT reagent Kit (TAKARA, Japan). Real-time quantitative PCR (qPCR) was performed using SYBR Green (YEASEN, China) according to the manufacturer’s instructions. The primers included the following: Sema3F forward 5′-TCAACAAGTGGAGCACATTC-3′, reverse 5′-ACAGTGGTGAGGCGGTAG-3′; Npn-2 forward 5′-TGGATGTACGACCGTGCCAAGTGG-3′, reverse 5′-CTGATACTCCATGTCATAG CTGGG-3′; actin forward 5′-ACCCCGTGCTGCTGACCGAG-3′, reverse 5′-TCCCGGCCAGCCAGGTCCA-3′. The relative content was examined using the 2^−ΔΔCq^ method [21].

### Western Blot

Protein extracts were separated by sodium dodecyl sulfate–polyacrylamide gel electrophoresis (SDS-PAGE) and then transferred to polyvinylidene fluoride (PVDF) membrane, blocked for 60 min, incubated with primary antibodies including rabbit anti-semaphorin3F (1:1000, Sigma, USA), rabbit anti-neuropilin-2 (1:1000, CST, USA), rabbit anti-neuropilin-2 (1:1000, Affinity Biosciences, USA), rabbit anti-Flag (1:1000, Affinity Biosciences, USA), rabbit anti-CRMP2 (1:10,000, Abcam, USA), p-T514-CRMP2 (1:1000, Affinity Biosciences, USA), p-S522-CRMP2 (1:1000, ECM Biosciences, USA), and p-T555-CRMP2 (1:1000, EM Biosciences, USA) at 4 °C overnight and incubated with secondary antibodies for 1 h. The bands of target proteins were analyzed with Clinx Image software (China). The optical density (OD) value was measured using ImageJ.

### Tissue Preparation

Rats were deeply anesthetized with 10% chloral hydrate (3.5 mL/kg, IP) and perfused trans-cardinally with normal saline 250 mL and 4% paraformaldehyde 250 mL for immunofluorescent staining. For the Timm stain, rats were perfused trans-cardinally with normal saline 250 mL, phosphate buffer solution (PBS) containing 0.1% sodium sulfide 250 mL, and 4% paraformaldehyde 250 mL in sequential. Brains were integrally removed and post-fixed in 4% paraformaldehyde at 4 °C overnight, gradually shifted to 10%, 20%, and 30% sucrose solution at 4 °C until sinking. Coronal slices of the dorsal hippocampus were prepared using a freezing microtome (CM1950, Leica, Heidelberg, Germany), stained immediately, or stored at – 80 ℃.

### Immunofluorescent Staining

After being treated with 0.1% triton for 15 min and blocked for 60 min, slices were incubated with primary antibodies including rabbit Npn-2 (1:100, CST, USA), mouse GFP (1:100, Affinity Biosciences, USA), rabbit GFP (1:100, Affinity Biosciences, USA), rabbit synaptoporin (1:100, Synaptic Systems, Germany), rabbit NeuN (1:100, Abcam, USA), and mouse GAD-65/67 (1:100, Abcam, USA) at 4 ℃ overnight and with secondary antibodies for 1 h at room temperature, then observed under a fluorescent microscope (Olympus BX51, Japan) or laser confocal fluorescence microscopy (Olympus FV3000, Japan).

### The Timm Stain

Slices were washed with PBS before incubating in the dark for 90 to 120 min in a 100-mL staining solution containing 2.55 g of citric acid, 2.35 g of sodium citrate, 0.85 g of hydroquinone, 5 mL of 17% AgNO3, and 60 mL of 50% gum arabic. When reaching satisfying staining, slices were terminated by immersing into PBS and sealed with Neutral balsam. MFS in the IML was estimated with the Timm score [22].

### Primary Hippocampal Neuron Culture

Primary hippocampal cultures were prepared from neonatal (P0) Sprague–Dawley rats as described previously [23]. Dissected in Hanks’ balanced salt solution, digested with papain with DNase for 20 min, and separated by gentle mechanical disruption, hippocampal neurons were seeded on poly-D-lysine (Sigma, USA) coated circular coverslips at 5 × 10^4^ cells per coverslip, or on coated 12-well plates at 1 × 10^6^ per well. About 2 h later, the medium was changed to Neurobasal-A medium (Gibco, USA) with 2% B27 supplement (Gibco, USA), 1% glutamine (Gibco, USA), and 1% penicillin/streptomycin (Gibco, USA). The cultures were maintained at 37 °C and 5% CO_2_.

### Secreted Sema3F Production

Alkaline phosphatase (AP)-tagged Sema3F (AP-Sema3F), AP-Sema3A, and AP ligands were generated as described previously [12]. Indicated plasmids were transfected into 293 T cells. The supernatant was collected and concentrated with Centricon filters (Millipore, USA). AP activity was measured by mixing AP-containing supernatant with 2X AP substrate buffer (15 mL of diethanolamine, pH = 9.8, containing 100 mg of p-nitrophenyl phosphate, 15 μL of 1 M MgCl_2_) and the OD 405 nm was read with a spectrophotometer.

### Immunocytochemistry and Axon Branch Analysis

Fixed with 4% paraformaldehyde for 15 min, treated with 0.1% triton for 5 min, and blocked for 60 min, neurons were incubated overnight at 4 °C with the primary antibodies including rabbit GFP (1:500, Affinity Biosciences, USA), rabbit mCherry (1:500, CST, USA), and mouse Tau-1 (1:100, Santa Cruz, USA) and with secondary antibodies for 1 h.

Primary cultures were then observed under a fluorescent microscope (Olympus BX51, Japan). The following parameters were analyzed using ImageJ: (1) the main axon refers to the longest one and its length was measured from the cell body to growth cone and (2) axon collaterals longer than 10 μm were traced, and their numbers and length were recorded. The experiment was repeated 3 times and about 20 neurons in each condition were analyzed.

### Statistical Analysis

Comparisons between two groups were performed using the unpaired *t*-test. For comparisons among multiple groups, a one-way analysis of variance (ANOVA) test plus a post hoc Tukey test was adopted. *P* < 0.05 was considered to be statistically significant. The data were expressed as mean ± SEM. GraphPad Prism 8.0 software was used for statistical analysis.

## Results

### Npn-2 Expression in Adult Hippocampus

**Fig. 1:**
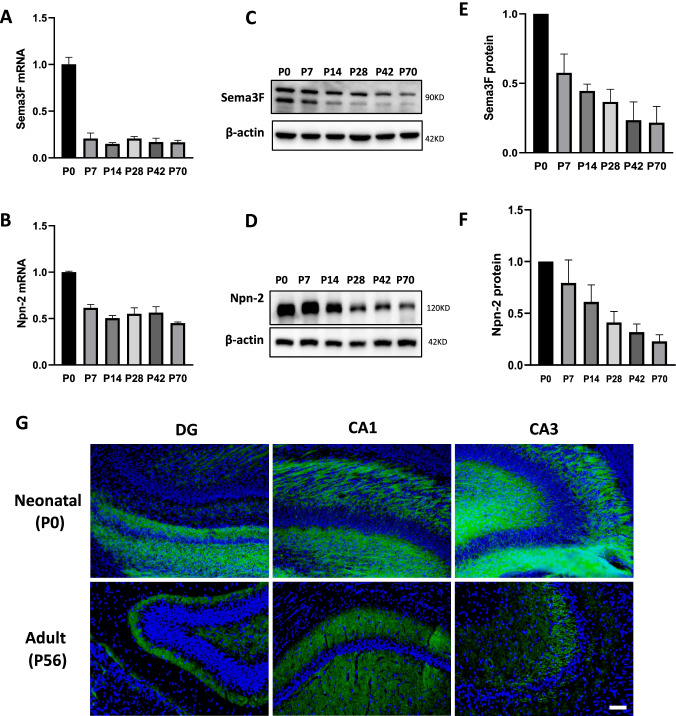
Expression of Npn-2 in adult rat hippocampus. **A**, **B** The mRNA levels of Npn-2 and its ligand Sema3F in rat hippocampus at different developmental stages (P0, P7, P14, P28, P42, and P70) were measured by real-time quantitative PCR. Npn-2 and Sema3F mRNA maintained their expression in adult hippocampus. **C**, **D** The protein levels of Sema3F and Npn-2 in rat hippocampus at different developmental stages (P0, P7, P14, P28, P42, and P70) were assessed by western blot analysis. Npn-2 and Sema3F retained their expression in adult hippocampus. **E**, **F** Quantitation of **C** and **D**, respectively. **G** Immunofluorescent detection of Npn-2 expression in rat hippocampus. Immunofluorescent staining with Npn-2 (green) and DAPI (blue) showed that Npn-2 is expressed in adult hippocampus, mainly in the neuropil, dentate gyrus granule cell inner molecular layer, and mossy fibers. Scale bar, 100 μm

Previous studies of Npn-2 signaling mainly focused on its functions during development. To understand its functions in adult animals, we first examined Npn-2 and its ligand Sema3F expression levels in rat hippocampus during different developmental stages. qPCR and western blot results showed that Npn-2 and Sema3F maintained their expression in adult hippocampus at both mRNA (Fig. 1A, B) and protein levels (Fig. 1C–F). Immunofluorescent staining with Npn-2 (green) antibody and DAPI (blue) revealed that Npn-2 in adult hippocampus is mainly distributed in the neuropil areas, specifically in dentate gyrus granule cell inner molecular layer and mossy fibers (Fig. 1G). Given its expression and distribution patterns in adult hippocampus, Npn-2 might play a role in the mature central nervous system.

### Npn-2 Knockdown Increases Spontaneous MFS

The key feature of MFS is the aberrant axon sprouting and abnormal orientation of dentate gyrus granule neurons, which could be regulated by axon guidance cues. Axon guidance cue receptor Npn-2 was found to distribute in dentate gyrus granule neurons in adult animals. We ask if Npn-2 signaling could potentially modulate MFS.

**Fig. 2:**
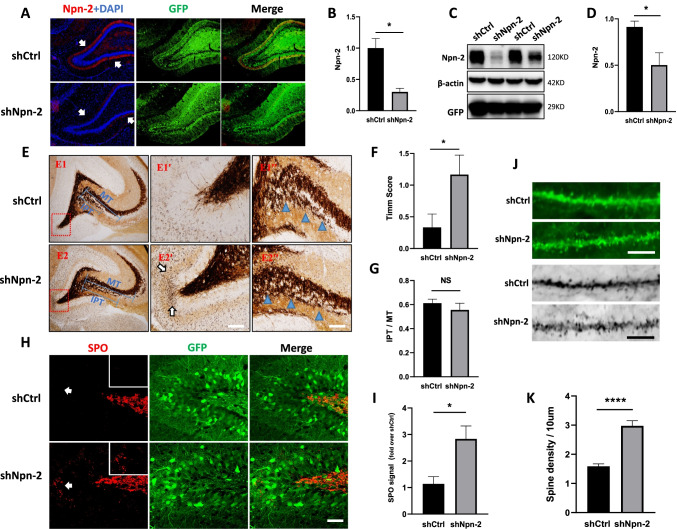
Increased spontaneous MFS in Npn-2 knockdown animals. **A** In vivo Npn-2 knockdown validated by immunofluorescent staining. Fourteen days after intrahippocampal injection of shCtrl AAV or shNpn-2 AAV, immunofluorescent staining with a Npn-2 antibody showed that Npn-2 expression in knockdown animals was significantly reduced compared with the controls. Arrows indicate Npn-2 (red). Scale bar: 100 μm. **B** Quantitation of immunofluorescence signal in **A**. *n* = 3, *P = 0.0128. **C** In vivo Npn-2 knockdown validated by western blot. Fourteen days after intrahippocampal injection with shCtrl AAV or shNpn-2 AAV, western blot results showed that endogenous Npn-2 in hippocampus was significantly reduced. **D** Quantitation of Npn-2 in **C**. *n* = 3, *P = 0.0484. **E** Spontaneous MFS in Npn-2 knockdown rats assessed by the Timm stain. Representative Timm stain images of rat brain coronal sections from either shCtrl- or shNpn-2 AAV-injected groups. Significantly increased MFS was detected in the shNpn-2 AAV-injected DG inner molecular layer (**E2**, E**2'**) when compared with shCtrl AAV-injected group (**E1**, **E1'**). No obvious difference in infrapyramidal tract (IPT) between shNpn-2 group and shCtrl group was observed (**E1''** vs **E2''**). **E1'**, **E2'**, **E1''**, and **E2''** are higher magnification views of E1 and E2, respectively. **E1**, **E2** Scale bar, 400 µm. **E1'**, **E2'** Scale bar, 100 µm. **E1''**, **E2''** Scale bar, 200 µm. **F** Quantitation of the Timm score in **E**. *n* = 6, *P = 0.0493. **G** Quantitation ratio of IPT length to MT length in **E**, *n* = 6, *P* > 0.05. **H** Spontaneous MFS in Npn-2 knockdown rats measured by immunofluorescent staining. Representative immunofluorescent images of rat brain coronal sections from either shCtrl- or shNpn-2 AAV-injected groups, labeled with synaptoporin (SPO) antibody and visualized under a laser scanning confocal microscope. A significantly increased SPO-positive puncta (red signal) number was detected in the shNpn-2-injected DG inner molecular layer when compared with shCtrl-injected group. Scale bar, 20 µm. **I** Quantitation of SPO punctate number in **H**, *n* = 3, *P = 0.0389. **J** Increased dendrite spine density in shNpn-2 granule neurons. Representative immunofluorescent images of rat brain coronal sections from either shCtrl- or shNpn-2 AAV-injected groups, labeled with GFP antibody, and visualized under a laser scanning confocal microscope. Significantly increased dendritic spine density was detected in shNpn-2-injected granule cells when compared with shCtrl-injected group. Scale bar, 10 µm. **K** Quantitation of spine density in **J**, ****P < 0.0001. Unpaired *t*-test. Error bars represent SEM

AAV encoding either control short hairpin RNA (shCtrl) or short hairpin RNA targeting rat Npn-2 (shNpn-2) was injected into the dentate gyrus of adult animals to reduce Npn-2 expression (Fig. 2A–D). Mossy fibers can be visualized with Timm stain. The Timm stain reaction deposits were rarely observed in the IML of control rats while Npn-2 knockdown significantly increased the reaction deposits of these animals (Fig. 2E, F). Synaptoporin (SPO) is known to specifically label mossy fiber synapses [24], thus, immunofluorescent staining with SPO was performed as a second method to verify the sprouting of mossy fibers. The result showed an increased number of SPO-positive puncta in the IML of Npn-2 knockdown dentate gyrus (Fig. 2H, I), suggesting that Npn-2 knockdown increased spontaneous MFS.

Sprouted mossy fibers synapse with dendritic spines of granule cells, thus forming excitatory neural circuits [25]. Embryonic Npn-2 knockout led to increased dendritic spine density [11]. We are curious about the functions of Npn-2 signaling in spine remodeling in adulthood. Immunofluorescent staining with GFP showed increased spine density in Npn-2 knockdown dentate granule neurons compared with control neurons (Fig. 2J, K).

Sema3F/Npn-2 signaling was reported to involve in axon pruning [26] during development. We are wondering if Npn-2 knockdown in adult brains could affect already pruned axons. Thus, we checked the ratio of infrapyramidal tract (IPT) length to main tract (MT) length in the adult hippocampus using the Timm stain. No obvious IPT defects were observed in Npn-2 knockdown animals (Fig. 2E, G).

### Reduction of Npn-2 Increases MFS in Epileptic Rats

**Fig. 3:**
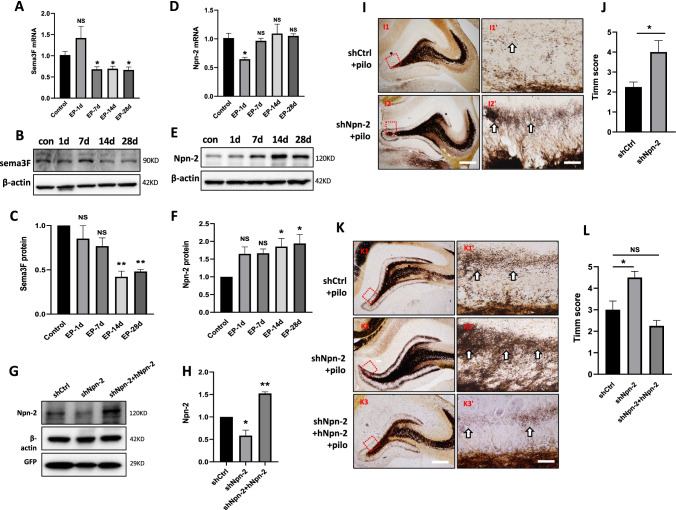
Increased MFS in Npn-2 knockdown epileptic rats. Sema3F mRNA level was decreased 7 days, 14 days, and 28 days after pilocarpine injection, measured by real-time PCR, *n* = 3, *P < 0.05. Sema3F protein level was decreased 14 days and 28 days after pilocarpine injection, measured by western blot. **C** Quantitation of Sema3F protein levels in **B**, *n* = 3, **P < 0.01. **D** Npn-2 mRNA level was transiently decreased 1 day after pilocarpine injection and recovered at 7 days, 14 days, and 28 days, measured by real-time PCR, *n* = 3, *P < 0.05. **E** Npn-2 protein level was increased 14 days and 28 days after pilocarpine injection, measured by western blot. **F** Quantitation of Npn-2 protein levels in **E**, *n* = 3, *P < 0.05. **G** Validation of Npn-2 knockdown and human Npn-2 expression in vivo. Western blot using an antibody recognizing both rat and human forms of Npn-2 showed that shNpn-2 AAV effectively reduced endogenous Npn-2 expression in vivo, which was rescued by hNpn-2 expression. **H** Quantitation of Npn-2 protein levels in **G**. *n* = 3, *P < 0.05, **P < 0.01. **I** MFS in epileptic rats at 14 days after pilocarpine injection. Representative Timm stain of rat brain coronal sections in shCtrl group and shNpn-2 group at 14 days after pilocarpine injection. Significantly increased MFS was observed in Npn-2 knockdown animals (**I2**, **I2'**) compared with control animals (**I1**, **I1'**). **I1'**–**I2'** are higher magnification views of **I1**–**I2**, respectively. **I1**–**I2** Scale bar, 400 µm. **I1'**–**I2'** Scale bar, 100 µm. **J** Quantitation of Timm’s score in **I**, unpaired *t*-test, and error bars represent SEM. *n* = 4, *P = 0.0272. **K** MFS in epileptic rats at 21 days after pilocarpine injection. Representative Timm stain of coronal brain sections in shCtrl group and shNpn-2 group at 21 days after pilocarpine intraperitoneal injection. Increased MFS was observed in shNpn-2 group (**K2**, **K2'**) compared with shCtrl group (**K1**, **K1'**), which was rescued by hNpn-2 expression (**K3**, **K3'**). **K1'**–**K3'** are higher magnification views of **K1**–**K3**, respectively. **K1**–**K3** Scale bar, 400 µm. **K1'**–**K3'** Scale bar, 100 µm. **L** Quantitation of Timm score in **K**, *n* = 4, *P = 0.0232. One-way ANOVA, post hoc Tukey test. Error bars represent SEM

Mutant animal results suggested that Sema3F and its receptor Npn-2 play important roles in animal models of epilepsy [11, 13]. We are wondering if the expression level of Sema3F and Npn-2 could change during epilepsy. To this end, we generated a pilocarpine-induced rat model of epilepsy. Hippocampal Sema3F mRNA and protein level was decreased in epileptic rats (Fig. 3A–C). On the other hand, the Npn-2 protein level was slightly increased in the hippocampus of epileptic rats (Fig. 3D, F), which could be the result of compensation for the reduced level of Sema3F. These data indicated that Sema3F and Npn-2 probably play roles in the pilocarpine-induced rat model of epilepsy.

MFS is proposed to relate to the recurrent excitation in the hippocampus during epileptogenesis [6, 27]. We found that Npn-2 knockdown could lead to spontaneous MSF. It is plausible that Npn-2 signaling is also involved in MFS during epilepsy. We then investigated the function of Npn-2 signaling in MFS in epileptic rats. Intrahippocampal injection of shCtrl AAV, shNpn-2 AAV, or shNpn-2 AAV plus hNpn-2 lentivirus was performed to regulate the level of Npn-2 (Fig. 3G, H). Fourteen days after intrahippocampal injection, a pilocarpine-induced rat model of epilepsy was established. Mossy fiber terminals were visualized at 14 days and 21 days after pilocarpine injection. Mossy fibers from Npn-2 knockdown animals had significantly more aberrant sprouting than control animals (for 14 days, Fig. 3I, J; for 21 days, Fig. 3K, L). The Timm stain at 21 days showed that hNpn-2 expression rescued the MFS phenotype (Fig. 3K, L). These data demonstrated that Npn-2 knockdown increased MFS of pilocarpine-induced rat model of epilepsy.

### Npn-2 Knockdown in Adult Hippocampus Increases Seizure Activity

Embryonic ablation of Npn-2 increased seizure activity in kainic acid-induced and pentylenetetrazol (PTZ) kindling seizure models [11, 14]. Given that Npn-2 expression persists in adult brains, especially in the hippocampus and the expression of Sema3F and Npn-2 change in mTLE patients and adult animal models of epilepsy [15–17], we ask whether it plays any role in epilepsy during adulthood.

**Fig. 4:**
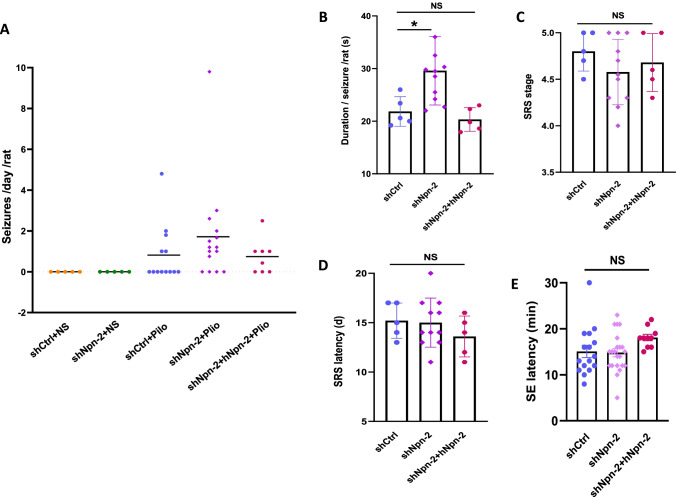
Npn-2 knockdown increases seizure activity. **A** Overview of SRS. No SRS was found with normal saline (NS) injection in both shCtrl and shNpn-2 groups. In pilocarpine injection groups, 5 out of 13 (38%) rats in shCtrl group and 11 out of 15 (73%) rats in shNpn-2 group developed SRS during the 21-day surveillance period after pilocarpine injection. Five out of 8 (62%) rats developed SRS in shNpn-2 plus hNpn-2 group. **B** Seizure duration. The mean duration per seizure in Npn-2 knockdown group was 29.59 s, which was significantly more than the control group (21.84 s, *P = 0.0307). hNpn-2 expression decreased seizure duration to control level (20.32 s, *P* > 0.05). **C** SRS stage. No significant difference in SRS stage was observed among shCtrl, shNpn-2, and hNpn-2 rescue groups (4.800 vs 4.577 vs 4.680, *P* = 0.4354). **D** SRS latency. There was no significant difference in SRS latency among shCtrl group, shNpn-2 group, and hNpn-2 rescue group (15.20 days vs 15.00 days vs 13.60 days, *P* = 0.4602). **E** SE latency. The SE latency of rats in shCtrl group, shNpn-2 group, and hNpn-2 rescue group were similar to each other (15.06 min vs 14.82 min vs 18.10 min, *P* = 0.1259). One-way ANOVA, post hoc Tukey test. Error bars represent SEM

Pilocarpine was injected to induce SE after 14 days and rats were then continuously surveilled for 21 days. Two out of 18 rats failed to develop SE in the shCtrl group. Three of 18 rats in the shCtrl group and 5 out of 20 rats in the shNpn-2 group died during the SRS stage (mortality rate: 16.7% in the shCtrl group and 25% in the shNpn-2 group). Behaviors of 13 rats in the shCtrl group and 15 rats in the shNpn-2 group were analyzed. Thirty-eight percent of rats in the shCtrl group while 73% in the shNpn-2 knockdown group developed SRS during the 21-day surveillance period (Fig. 4A). The mean duration per seizure of Npn-2 knockdown rats was 29.59 s, which was significantly more than the 21.84 s of control rats (Fig. 4B). There was no significant difference in SRS stage (Fig. 4C), SRS latency (Fig. 4D), and SE latency (Fig. 4E) between shCtrl group and shNpn-2 group. The data indicated that reduction of Npn-2 increased seizure activity in pilocarpine-induced rat model.

In human Npn-2 (hNpn-2) rescue group, 2 rats died during SRS (mortality rate: 20%). Behaviors of 8 rats were analyzed. Fewer rats in the rescue group (62.5%) developed SRS when compared with shNpn-2 group (Fig. 4A). The mean SRS duration in the rescue group was similar to that in shCtrl group (Fig. 4B). These data suggested that the expression of hNpn-2 ameliorated the increased seizure activity caused by Npn-2 knockdown, suggesting the specificity of Npn-2 to modulate seizure activity in adult.

### Npn-2 Knockdown in Adult Has No Effect on GABAergic Interneurons Survival

Previous studies mainly focused on the effect of Npn-2 signaling in the development stages and their data have shown that embryonic Npn-2 knockout increased seizure activity by reducing the number of GABAergic interneurons [11]. We examined whether Npn-2 knockdown in adult animals affects interneuron survival.

Immunofluorescent staining with NeuN showed no loss of neurons in general (Figure S2A and S2B), and labeling with GAD-65/57 showed no obvious GABAergic interneuron loss in Npn-2 knockdown rats (Figure S2C and S2D). These results suggested that Npn-2 knockdown in adult hippocampus influences seizure activity without affecting GABAergic inhibitory interneurons, which was partly different from that during development.

### Npn-2 Signaling Controls Axon Collateral Formation

Given the 3-step cellular mechanism of MFS, namely axon branching, reverse projection, and fasciculation [7], we tested the role of Npn-2 signaling in axon collateral formation and outgrowth using an in vitro assay.

**Fig. 5:**
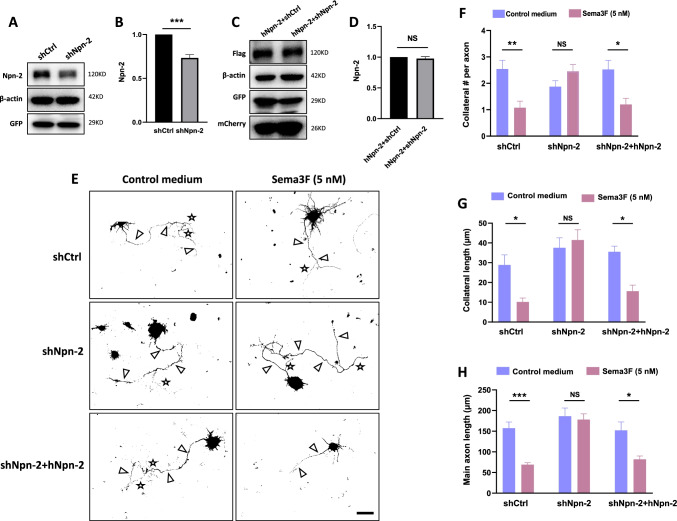
Npn-2 controls axon collateral formation. **A** Npn-2 knockdown by shNpn-2 validated in primary neuron cultures by western blot using an antibody for rat Npn-2. **B** Quantitation of Npn-2 in **A**, *n* = 3, ***P = 0.0003. **C** hNpn-2 expression validated by western blot. Expression of hNpn-2 in neurons was detected by western blot using an anti-Flag antibody, showing that the expression of human full-length Npn-2 cannot be affected by the rat shNpn-2 used. **D** Quantitation of Npn-2 in **C**, *n* = 3, *P* > 0.05. **E** Neonatal rat primary hippocampal neurons were transfected with shCtrl, shNpn-2, or shNpn-2 plus hNpn-2 plasmids at DIV 0 and treated with either control medium or Sema3F (5 nM) at 48 h after transfection for 24 h. Axons and their collaterals were detected by immunofluorescent staining with GFP and Tau-1. Arrows indicate main axons and stars indicate axon collaterals. Scale bar, 40 μm. **F**, **G** Quantitation of axon collateral number and length in **E**. Sema3F treatment reduced the number and length of axon collaterals in neurons transfected with shCtrl but not in neurons with shNpn-2 transfection, which was rescued by hNpn-2 co-transfection. *P < 0.05, **P < 0.01. **H** Quantitation of main axon length in **E**. Main axon elongation in neurons transfected with shCtrl was inhibited upon Sema3F treatment, but not in neurons with shNpn-2 transfection, which was rescued by hNpn-2 expression. **P < 0.01, ***P < 0.001. One-way ANOVA, post hoc Tukey test. Error bars represent SEM

Dissociated hippocampal cultures were transfected with shCtrl, shNpn-2, or shNpn-2 plus hNpn-2 plasmids at 0 day in vitro (DIV 0). Npn-2 knockdown by shNpn-2 and hNpn-2 expression were validated by in vitro experiments (Fig. 5A–D, Figure S1). Forty-eight hours after transfection, primary cultures were treated with a control medium or 5 nM AP-Sema3F for 24 h. Axons and their collaterals were labeled with GFP and Tau-1, a biomarker for axons (Fig. 5E, Figure S3). Sema3F treatment reduced the number and length of axon collaterals as well as the main axon length of shCtrl-transfected neurons but not Npn-2 knockdown neurons. These phenotypes were rescued by hNpn-2 expression (Fig. 5F–H). The results suggested that Sema3F modulates axon collateral formation and elongation through Npn-2, which may serve as its cellular mechanism to regulate MFS.

### Npn-2 Signaling Regulates CRMP2 Phosphorylation

CRMP2 is a classical downstream molecule in secreted semaphorin signaling [25, 26]. CRMP2 exhibits neurite outgrowth-promoting function, which is regulated by its phosphorylation state [28]. Our previous data have shown that the phosphorylation of CRMP2 at serine 522 (S522), threonine 514 (T514), or threonine 555 (T555) was downregulated in the hippocampus of pilocarpine-induced rat model of epilepsy at 1 day, 7 days, and 14 days after pilocarpine injection [29], suggesting that CRMP2 may play some roles in epilepsy. Another semaphorin, Sema3A, is able to increase CRMP2 phosphorylation [30]. Here, in vitro and in vivo assays were performed to examine if Sema3F/Npn-2 signaling regulates CRMP2 phosphorylation.

**Fig. 6:**
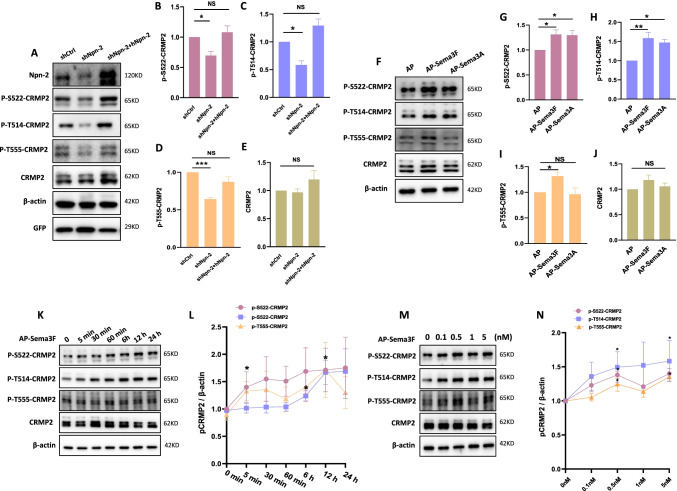
Sema3F/Npn-2 signaling regulates CRMP2 phosphorylation. **A** Reduction of CRMP2 phosphorylation in Npn-2 knockdown tissues. Adult rat hippocampi injected with various viruses as indicated were subjected to western blot with different phospho-CRMP2 antibodies. The levels of p-S522, p-T514, and p-T555-CRMP2 were significantly reduced in Npn-2 knockdown animals compared with the control group while the expression of CRMP2 was not changed, and the knockdown effects were rescued by the expression of hNpn-2. **B**–**E** Quantitation of **A**. *n* = 4, *P < 0.05, ***P < 0.001. **F** Semaphorin induced CRMP2 phosphorylation in primary cultures. Primary cultured neurons were treated with 5 nM AP, AP-Sema3F, or AP-Sema3A for 12 h. CRMP2 phosphorylation levels were detected by various phospho-CRMP2 antibodies as indicated using western blot. Sema3F treatment upregulated the levels of p-S522, p-T514, and p-T555-CRMP2 while made no effect on CRMP2 expression. **G**–**J** Quantitation of **F**. *n* = 4, *P < 0.05, **P < 0.01. One-way ANOVA, post hoc Tukey test. Error bars represent SEM. **K** Time course of Sema3F regulating CRMP2 phosphorylation. Primary cultured neurons were treated with 5 nM AP-Sema3F for 5 min, 30 min, 60 min, 6 h, 12 h, and 24 h. Levels of CRMP2 phosphorylation were detected by various phospho-CRMP2 antibodies as indicated using western blot. The levels of p-S522-CRMP2, p-T514-CRMP2, and p-T555-CRMP2 increased over time and peaked at around 12 h, while CRMP2 expression was not significantly changed. **L** Quantitation of **K** (*n* = 3). **M** Sema3F that regulates CRMP2 phosphorylation is dose-dependent. Primary cultured neurons were treated with AP-Sema3F at various doses as indicated for 12 h. CRMP2 phosphorylation levels were detected by phospho-CRMP2 antibodies as indicated using western blot. The levels of p-S522-CRMP2, p-T514-CRMP2, and p-T555-CRMP2 were increased over increased doses and peaked around 0.5 nM, while the expression of CRMP2 was not significantly changed. **N** Quantitation of **M** (*n* = 3)

Rats with shCtrl AAV, shNpn-2 AAV, or shNpn-2 AAV plus hNpn-2 lentivirus injection were sacrificed 14 days after injection for western blot. Npn-2 knockdown reduced CRMP2 phosphorylation levels at S522, T514, or T555 sites, but not the total CRMP2 amount. The reduction of CRMP2 phosphorylation was rescued by hNpn-2 expression (Fig. 6A–E).

Primary neuronal cultures were treated with 5 nM AP, AP-Sema3F, or AP-Sema3A for 12 h. Western blot results showed that CRMP2 can be phosphorylated by Sema3F while levels of total CRMP2 remained unchanged (Fig. 6F–J). Time-course and dose-dependent experiments showed that CRMP2 phosphorylation was increased over time (peaked at 12 h) (Fig. 6K, L) and dose (peaked at 0.5 nM) (Fig. 6M, N), demonstrating the specificity of Sema3F/Npn-2 signaling in regulating CRMP2 phosphorylation.

Our data indicated that Sema3F/Npn-2 signaling facilitated CRMP2 phosphorylation, which lays the foundation for this pathway to regulate the function of CRMP2.

### CRMP2 Mediates Npn-2 Controlled Axon Collateral Formation

CRMP2 was reported to mediate Sema3F/Npn-2 signaling in axon retraction, axon guidance, axon pruning, and dendritic spine remodeling [26]. In addition, the axon outgrowth promoting function of CRMP2 is mainly determined by its phosphorylation state, which was validated to be regulated by Sema3F/Npn-2 signaling in this study. Here we asked whether Npn-2 signaling regulates axon collateral formation via CRMP2.

**Fig. 7:**
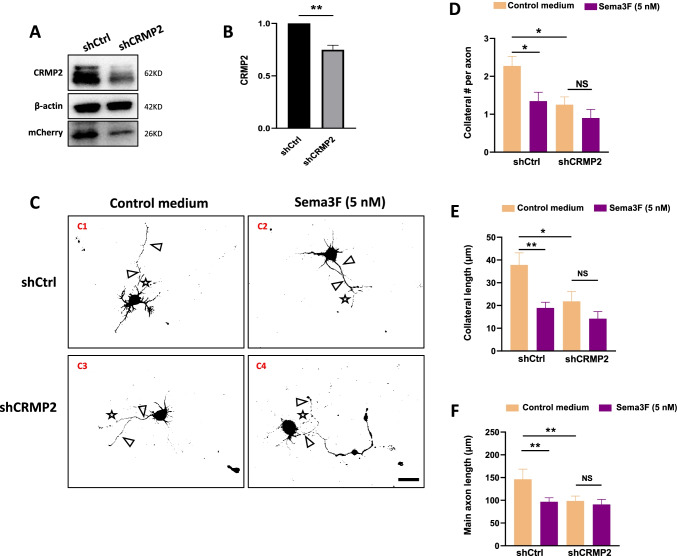
Npn-2 signaling regulates axon collateral formation through CRMP2. **A** CRMP2 knockdown by CRMP2 shRNA (shCRMP2) was validated in primary neurons by western blot. **B** Quantitation of CRMP2 in *n* = 3, **P = 0.0045. (C) CRMP2-mediated Sema3F controlled collateral formation. Neonatal rat primary hippocampal neurons were transfected with shCtrl or shCRMP2 plasmids at DIV 0. Forty-eight hours after transfection, the cultures were treated with a control medium or 5 nM AP-Sema3F for 24 h. Main axons and their collaterals were detected by immunofluorescent staining with mCherry and Tau-1. Arrows indicate main axons and stars indicate axon collaterals. Scale bar, 40 µm. **D**, **E** Quantitation of axon collateral number and length in **C**. CRMP2 knockdown led to a decrease in the number and length of axon collaterals. Sema3F treatment reduced the number and length of axon branches in neurons transfected with shCtrl but not in neurons with shCRMP2 transfection. *P < 0.05, **P < 0.01. **F** Quantitation of main axon length in **C**. CRMP2 knockdown led to a decrease in main axon length. Sema3F treatment reduced the length of main axons in neurons transfected with shCtrl but not in neurons with shCRMP2 transfection. **P < 0.01. One-way ANOVA, post hoc Tukey test. Error bars represent SEM

Dissociated hippocampal cultures were transfected with shCtrl or CRMP2 shRNA (shCRMP2) plasmids at DIV 0. CRMP2 knockdown by shCRMP2 has been validated in vitro (Fig. 7A, B, Figure S1). Cultures were treated with a control medium or 5 nM AP-Sema3F at 48 h after transfection for 24 h. The axons and their collaterals were labeled with Tau-1 and mCherry antibodies (Fig. 7C, Figure S4). Compared with the control medium, Sema3F treatment decreased axon collateral number and length as well as main axon length in shCtrl-transfected neurons (Fig. 7D–F). In control medium-treated neurons, CRMP2 knockdown reduced axon branch number and length as well as main axon length compared with shCtrl transfection (Fig. 7D–F). Interestingly, upon CRMP2 was knocked down, Sema3F failed to inhibit axon collateral number and length as well as main axon length (Fig. 7D–F). These results demonstrated that CRMP2 mediates Sema3F/Npn-2 controlled axon collateral formation and elongation.

### Npn-2 Modulates MFS Through CRMP2

We have demonstrated that Sema3F/Npn-2 signaling modulates axon collateral formation through CRMP2. Since axon branching is the first step of MFS, we then test if CRMP2 mediates Npn-2 signaling in MFS in epileptic animals.

**Fig. 8:**
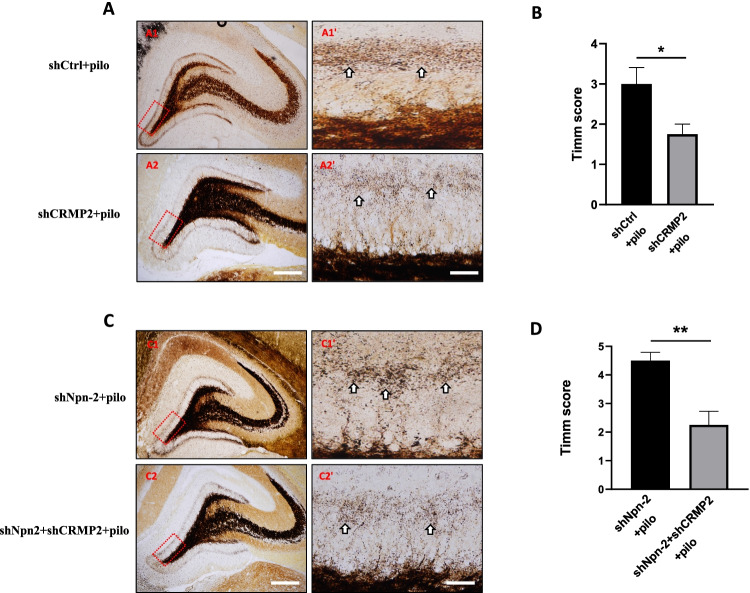
Npn-2 modulates MFS through CRMP2. **A** CRMP2 knockdown reduced MFS in epilepsy animals. Representative Timm stain of rat brain coronal sections in various AAV-injected groups as indicated at 21 days after pilocarpine injection. CRMP2 knockdown in epileptic rats (**A2**, **A2'**) reduced MFS compared with the control group (**A1**, **A1'**). **A1'**–**A2'** are higher magnification views of **A1**–**A2**, respectively. **A1**–**A2** Scale bars: 400 µm. **A1'**–**A2'** Scale bars: 100 μm. **B** Quantitation of Timm score in **A**, *n* = 4, *P = 0.0401. **C** CRMP2 mediated Npn-2 signaling in MFS in epilepsy animals. Representative Timm stain of rat brain coronal sections in various AAV-injected groups as indicated 21 days after pilocarpine injection. Reduction of CRMP2 (**C2**, **C2'**) rescued the increased MFS caused by Npn-2 knockdown (**C1**, **C1'**). **C1'**–**C2'** are higher magnification views of **C1**–**C2**, respectively. **C1**–**C2** Scale bars: 400 µm. **C1'**–**C2'** Scale bars: 100 μm. **D** Quantitation of Timm score in **C**, *n* = 4, **P = 0.0069. Unpaired *t*-test. Error bars represent SEM

Rats were injected with shCtrl AAV, shCRMP2 AAV, shNpn-2 AAV, or shNpn-2 AAV plus shCRMP2 AAV 14 days before pilocarpine-induced rat model of epilepsy establishment. Epileptic rats were sacrificed at 21 days after pilocarpine injection to visualize hippocampal mossy fiber terminals using the Timm stain. CRMP2 knockdown epileptic animals showed significantly reduced MFS compared with control epileptic animals (Fig. 8A, B). Furthermore, the reduction of CRMP2 rescued the increased MFS in Npn-2 knockdown epileptic animals (Fig. 8C, D). These results clearly showed that CRMP2 is mediating Npn-2 signaling in regulating MFS.

### CRMP2 Mediates Npn-2 Function in Regulating Seizure Activity

Given that CRMP2 plays significant role in Npn-2 mediated MFS and MFS is closely related to seizure activity, we next asked whether Npn-2 signaling regulates seizure activty through CRMP2.

**Fig. 9:**
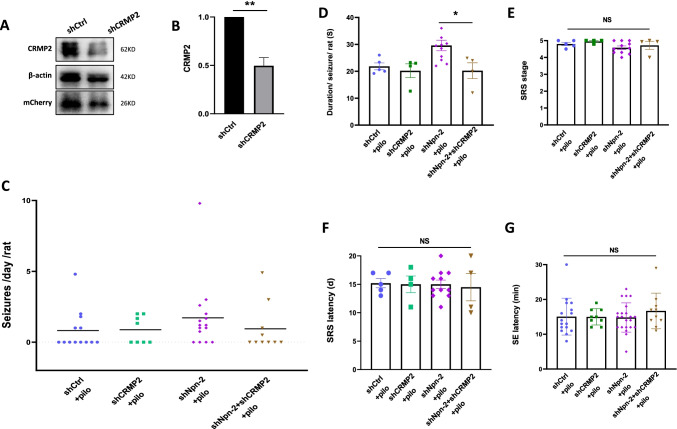
CRMP2 mediates Npn-2 function in regulating seizure activity. **A** CRMP2 knockdown validation in vivo. Fourteen days after intrahippocampal injection of shCtrl AAV or shCRMP2 AAV, rats were sacrificed for western blot analysis, and an effective knockdown of CRMP2 was observed. **B** Quantitation of CRMP2 in **A**, *n* = 3, **P = 0.0039. **C** Overview of SRS. Five out of 13 (38%) rats in shCtrl group and 4 out of 8 (50%) rats in shCRMP2 group developed SRS during the 21-day surveillance period post pilocarpine injection. Four out of 10 (40%) in shNpn-2 plus shCRMP2 group, while 11 out of 14 (73%) rats in shNpn-2 alone group developed SRS. **D** Mean duration per seizure. Seizure duration in shNpn-2 plus shCRMP2 group was 20.23 s, which was significantly less than the 29.59 s in shNpn-2 group (*P = 0.0459). **E** SRS stage. There was no significant difference in SRS stage among shCtrl group, shCRMP2 group, shNpn-2 group, and shNpn-2 plus shCRMP2 group (4.800 vs 4.950 vs 4.577 vs 4.718, P = 0.2579). **F** SRS latency. No significant difference in SRS latency was found among shCtrl group, shCRMP2 group, shNpn-2 group, and shNpn-2 plus shCRMP2 group (15.20 days vs 15.00 days vs 15.00 days vs 14.50 days, P = 0.9867). **G** SE latency. The SE latency of rats in among shCtrl group, shCRMP2 group, shNpn-2 group, and shNpn-2 plus shCRMP2 group was similar to each other (15.06 min vs 15.00 min vs 14.82 min vs 16.7 min, *P* = 0.7306). One-way ANOVA, post hoc Tukey test. Error bars represent SEM

To this end, rats with shCRMP2 AAV or shNpn-2 AAV plus shCRMP2 AAV were intraperitoneally injected with pilocarpine and surveilled for 21 days. The efficacy of shCRMP2 AAV was validated in vivo (Fig. 9A, B). Two of 10 rats in the shCRMP2 group died during SRS (mortality rate: 20%). Behavior data of 8 rats in the shCRMP2 group and 10 rats in shNpn-2 plus shCRMP2 group were analyzed.

Fewer rats in shNpn-2 plus shCRMP2 group (40%) developed SRS during 21-day surveillance compared with that in the shNpn-2 group (73%) (Fig. 9C). The mean duration per seizure in shNpn-2 plus shCRMP2 group was 20.23 s, which was significantly less than the 29.59 s in the shNpn-2 group (Fig. 9D). There was no significant difference in SRS stage (Fig. 9E), SRS latency (Fig. 9F), and SE latency (Fig. 9G) among groups. These data showed that increased seizure activity in Npn-2 knockdown rats can be rescued by the reduction of CRMP2 expression, suggesting that CRMP2 mediates Npn-2 signaling in regulating seizure activity.

## Discussion

In this study, we used a dentate gyrus-specific Npn-2 knockdown approach to study the function of Npn-2 in adult animals. Our results demonstrated that Npn-2 reduction increased seizure activity upon pilocarpine stimulation. Increased MFS and dendritic spine density were observed in Npn-2 knockdown hippocampus, suggesting more excitatory synapse formation. Npn-2 signaling was also demonstrated to regulate CRMP2 phosphorylation at S522, T514, and T555 sites. Reduction of CRMP2 expression rescued the increased SRS and MFS caused by Npn-2 knockdown in epileptic rats. Primary culture experiments illustrated that Npn-2 signals through CRMP2 to modulate axon collateral formation and elongation, which could be the underlying cellular mechanisms for MFS. Our work revealed a novel function of Npn-2 signaling in neural rewiring in adult brains.

### Npn-2 Signaling Modulates Seizure Activity in Adult by Regulating MFS

During the development stages, Sema3F and Npn-2 knockout mice showed increased seizure susceptibility and severity, which are suggested to be caused mainly by the reduced number of GABAergic inhibitory neurons [11, 13, 14]. We found that Npn-2 maintained its expression in the adult hippocampus, especially in dentate gyrus granule neurons. Also, the expression of Sema3F and Npn-2 changed significantly in mTLE patients [15] and animal models of epilepsy [16, 17]. It is plausible that Npn-2 signaling is important in epilepsy during adulthood as well. In this study, we found that Npn-2 knockdown in adult hippocampus led to higher SRS incidence and longer SRS duration in a pilocarpine-induced animal model of epilepsy, which was ameliorated by expression of human full-length Npn-2. It is unlikely that the effects of Npn-2 knockdown on seizure activity are due to the reduction in GABAergic interneurons since no obvious change in GABAergic interneuron number was observed in the adult Npn-2 knockdown animals. Our results suggested an additional mechanism for Npn-2 signaling in epileptogenesis in adult animals.

Along with increased seizure activity, we found increased MFS in Npn-2 knockdown rats. The process of MFS was proposed to include mossy fiber axon collateral formation, reverse projection, and fasciculation [7]. Proper outgrowth and pathfinding of mossy fibers were under precise regulation of diffusible chemoattractant [7, 31]. Sema3F/Npn-2 signaling was initially discovered as a repulsive axon guidance pathway [32]. Npn-2 was found to express in the adult hippocampal dentate gyrus, especially at mossy fibers and IML. Under normal conditions, Sema3F/Npn-2 signaling could function as the gatekeeper to restrain mossy fiber from sprouting into IML. With the loss of Npn-2 expression in granule neurons, mossy fibers would make aberrant branching and elongation, which leads to MFS.

The MFS hypothesis holds that sprouted terminals of excitatory granule cells innervate themselves by forming asymmetric synapses with their dendrites and dendritic spines, thus building up recurrent excitatory networks [31]. It is worth mentioning that we also observed increased dendritic spine density in Npn-2 knockdown granule cells in vivo. Our results suggest that Npn-2 knockdown results in robust MFS and synapse remodeling, constituting the structural basis for local excitatory networks. We also find that CRMP2 mediates Npn-2 signaling in MFS and epileptogenesis. A novel antiepileptic drug, lacosamide (Vimpat, LCM), was reported to exert both anti-seizure effects through interacting with voltage-gated sodium channel and anti-epileptogenesis effects through inhibiting CRMP2-mediated tubulin polymerization [33], which may serve as its mechanism to suppress MFS [33, 34]. Thus, LCM is regarded as the only anti-epileptogenesis drug at present. These data clearly indicated that disease-modifying therapies targeting MFS are promising in epilepsy treatment.

Another potential mechanism for epileptic seizures is the imbalance between inhibitory and excitatory neurons [35]. Glutamate receptors, especially AMPA receptors, were increased in epilepsy and regarded as one of the major drivers of seizures [36, 37]. Sema3F/Npn-2 signaling is essential for homeostatic downscaling of AMPA receptors responding to increased neuronal activity [12]. The loss of AMPA receptor homeostatic regulation in Npn-2 knockdown rats could also contribute to the increased seizure activity in adult epileptic animals.

### Candidate Molecules Modulating MFS in Npn-2 Signaling

We found that Sema3F/Npn-2 signaling phosphorylates CRMP2 at S522, T514, and T555 sites. The functions of CRMP2 can be modulated by its phosphorylation state [38]. Phosphorylated CRMP2 inhibits its interaction with tubulin heterodimers and negatively regulates microtubule assembly, which suppresses axonal outgrowth. In Npn-2 knockdown animals, it is probable that dephosphorylation of CRMP2 promotes microtubule assembly, and facilitates axon collateral formation and elongation, which ultimately leads to MFS.

Discovered as a regulator of growth cone collapse, CRMP2 involves neuronal polarity via binding to tubulin heterodimers, facilitating their transportation to the end of growing microtubule [39, 40] as well as enhancing the GTPase activity of the β-tubulin to promote tubulin heterodimer polymerization [41]. The level of CRMP2 phosphorylation was decreased in pilocarpine- or kainic acid-induced epilepsy models [34]. LCM was reported to prevent posttraumatic axon sprouting by inhibiting CRMP2-mediated tubulin polymerization and neurite outgrowth [33]. Therefore, CRMP2 could potentially involve in the process of epilepsy-induced MFS.

CRMP2 was reported to be phosphorylated through several pathways [30, 42, 43]. In Sema3A signaling, cyclin-dependent kinase (Cdk5) phosphorylates CRMP2 at S522 [30], priming CRMP2 for subsequent phosphorylation by glycogen synthase kinase-3β (GSK3β) at residues T509-T514-S518 [30, 42]. Sema3F is able to activate Cdk5 [44] and Gsk3β [45] in cultured hippocampal neurons. Sema3F likely phosphorylates CRMP2 at S522 and T514 similar to the way Sema3A phosphorylates it. Npn-2 is probably through RhoA to phosphorylate CRMP2 at the T555 site, since this site is a Rho kinase target and Npn-2 signaling can activate RhoA [43, 44].

MICAL family proteins were initially found as downstream molecules to mediate Sema3A and Sema3F-induced axonal repulsion [46]. In the autosomal-dominant lateral temporal epilepsy (ADLTE) family, two ADLTE-causing variants in the MICAL-1 gene were notified [47]. MICAL-1 expression was downregulated in TLE patients and a pilocarpine-induced rat model of epilepsy [48]. MICAL-1 flavoprotein monooxygenase activity causes actin filaments disassembly, thus regulating actin cytoskeleton organization in developing and mature neurons [47, 49]. These results suggest that MICAL family proteins may function as another potential downstream component to mediate Npn-2 in axon collateral formation and MFS by regulating actin cytoskeleton dynamics.

β-Chimaerin, a Rac GTPase activating protein, mediates Sema3F signaling in IPT pruning during development by binding to Npn-2 [50]. Chimaerins are involved in counteracting myelin-associated glycoprotein-induced neurite outgrowth inhibition [51]. Whether β-Chimaerin mediates Sema3F/Npn-2 signaling in axon collateral formation and outgrowth during MFS needs to be further elucidated.

### Working Model for Npn-2 Signaling in MFS and Seizure Activity

**Fig. 10:**
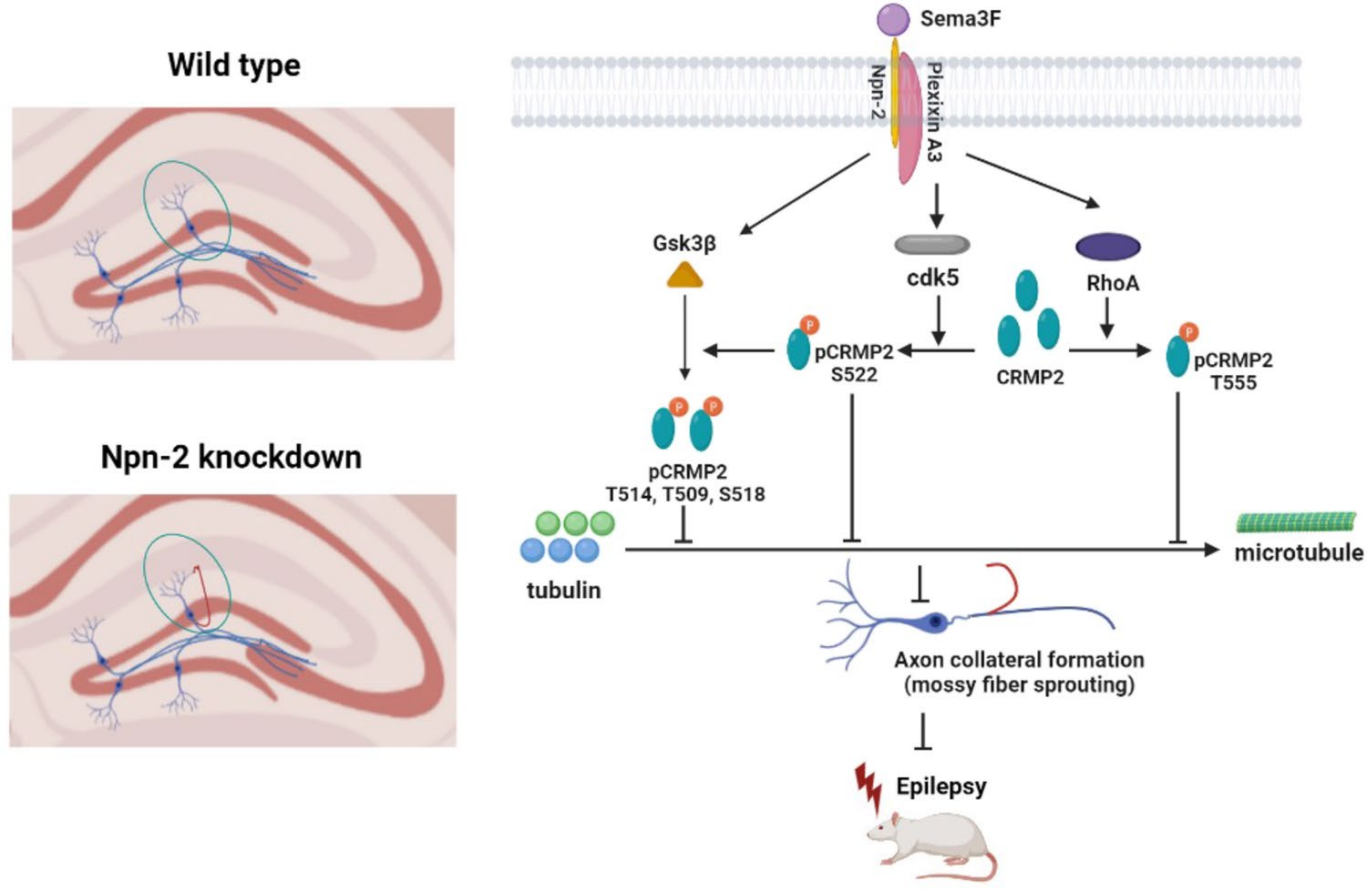
The schematic for Npn-2 in regulating seizure activity and MFS. A working model for Npn-2 signaling in MFS. Sema3F/Npn-2 signaling downregulates CRMP2 phosphorylation, thus diminishing the function of CRMP2 to promote axon collateral formation and elongation. MFS is restrained in this way and seizure activity of pilocarpine-induced rat model of epilepsy was then inhibited

We summarized a working model for Sema3F/Npn-2 signaling in modulating seizure activity and MFS in adult animals (Fig. 10). The holoreceptor complex of Npn-2 and plexin A3 transduces the extracellular signal of Sema3F into cells. Sema3F/Npn-2 signaling upregulates the phosphorylation of downstream molecule CRMP2 at S522, T514, and T555 sites, possibly through intermediate molecules like Cdk5, Gsk3β, and Rho-A [43–45]. The function of CRMP2 is mainly regulated by its phosphorylation state. Upon phosphorylation, the ability of CRMP2 to promote tubulin polymerization and microtubule assembly is weakened. Thereby, the formation, outgrowth, and aberrant projection of axon collaterals and main axon elongation are inhibited. Thus, the process of MFS is restrained by Sema3F/Npn-2 signaling, and seizure activity is therefore inhibited.

## Conclusions

We demonstrated that dentate gyrus-specific Npn-2 knockdown in adult brains increased seizure activity by regulating MFS. This is a distinct mechanism from that Npn-2 modulates seizure activity by regulating GABAergic interneuron migration during development. We have also uncovered its molecular and cellular mechanisms. Since the asymmetric synaptic connections between sprouted mossy fibers and dendritic spines constitute the structural basis for neural hyper synchronization and spontaneous recurrent seizures, disease-modifying therapies targeting signaling pathways regulating neural rewiring such as Sema3F/Npn-2 signaling are promising in treating epilepsy in adulthood.

## Supplementary Information

Below is the link to the electronic supplementary material.

Supplementary file1 (DOC 1515 KB)

## Data Availability

The datasets generated during the current study are available from the corresponding author on reasonable request.
