# Supplementary material for: Neuropilin-2 Signaling Modulates Mossy Fiber Sprouting by Regulating Axon Collateral Formation Through CRMP2 in a Rat Model of Epilepsy
Source: Mol Neurobiol. 2022 Aug 31;59(11):6817–33. doi: 10.1007/s12035-022-02995-0 (PMC9525442; doi:10.1007/s12035-022-02995-0)
Supplement: Supplementary file 1 — Supplementary file1 (DOC 1515 KB) [file 12035_2022_2995_MOESM1_ESM.doc]

**Supplementary Materials**

**Neuropillin-2 Signaling Modulates Mossy Fiber Sprouting by Regulating**

**Axon Collateral Formation through CRMP2 in a Rat Model of Epilepsy**

Yuxiang Li^1^, Fangchao Tong^1^, Yiying Zhang^1^, Yiying Cai^1^, Jing Ding^1^, Qiang Wang^1*^, Xin Wang^1,2*^

**Supplementary Materials**

**Figure S1. Validation of plasmids used in this study *in vitro*.**

**Figure S2. Npn-2 knockdown in adult hippocampus had no effect on GABAergic interneurons.**

**Figure S3. Npn-2 signaling controls axon collateral formation.**

**Figure S4. CRMP2 mediates Sema3F/Npn-2 signaling in axon collateral formation.**

**
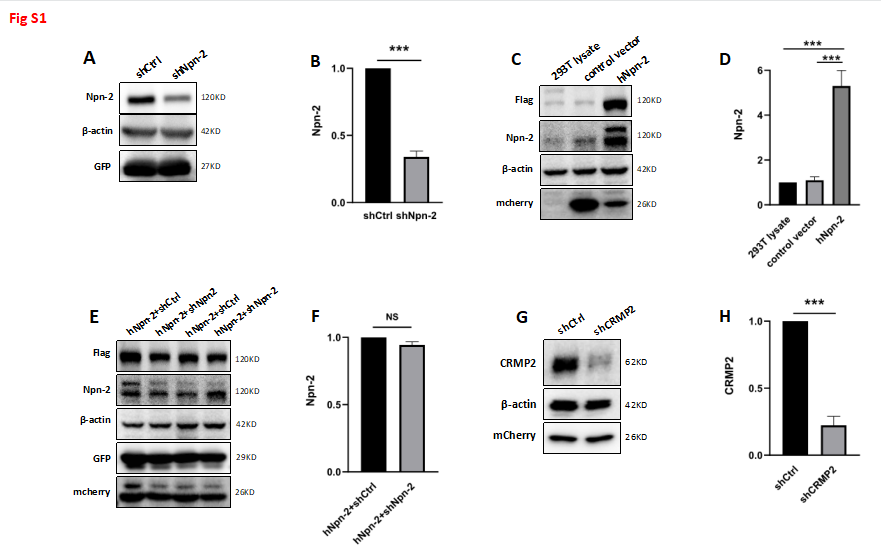
**

**Figure S1. Validation of plasmids used in this study *in vitro*.**

1. Npn-2 knockdown verified in 293T cells. 293T cells were transinfected with rat Npn-2

plus shCtrl plasmids or rat Npn-2 plus shNpn-2 plasmids using lipofectamine reagent 2000 and harvested after 48 hours. Western blot analysis showed that shNpn-2 plasmid effectively knocked down exogenous rat Npn-2.

1. Quantitation of Npn-2 in A, n=3, ***P=0.0001.

(C) Human Npn-2 expression validated in 293T cells. hNpn-2 plasmids were transfected into 293T cells, 48 hours after transfection, cell lysates were collected for western blot. hNpn-2 plasmid expressed human full-length Npn-2 in 293T cells was detected using an antibody recognizing both rat and human form of Npn-2 and anti-Flag antibody.

(D) Quantitation of Npn-2 in C, n=3, ***P<0.001

(E) hNpn-2 cannot be knocked down by rat shNpn-2. 293T cells were co-transfected with hNpn-2 plus shCtrl plasmids or hNpn-2 plus shNpn-2 plasmids, and harvested after 48 hours for western blot. The level of hNpn-2 was detected by either anti-Flag or anti Npn-2 antibodies. Human Npn-2 cannot be knocked down by rat shNpn-2.

(F) Quantitation of Npn-2 in E, P>0.05.

(G) Validation of CRMP2 knockdown in 293T cells. 293T cells were transfected with rat CRMP2 plus shCRMP2 plasmids or rat CRMP2 plus shCtrl plasmids using lipofectamine reagent 2000 and harvested after 48 hours. Western blot analysis showed that shCRMP2 plasmid effectively knocked down exogenous rat CRMP2.

(H) Quantitation of CRMP2 in G, n=3, ***P=0.0003.


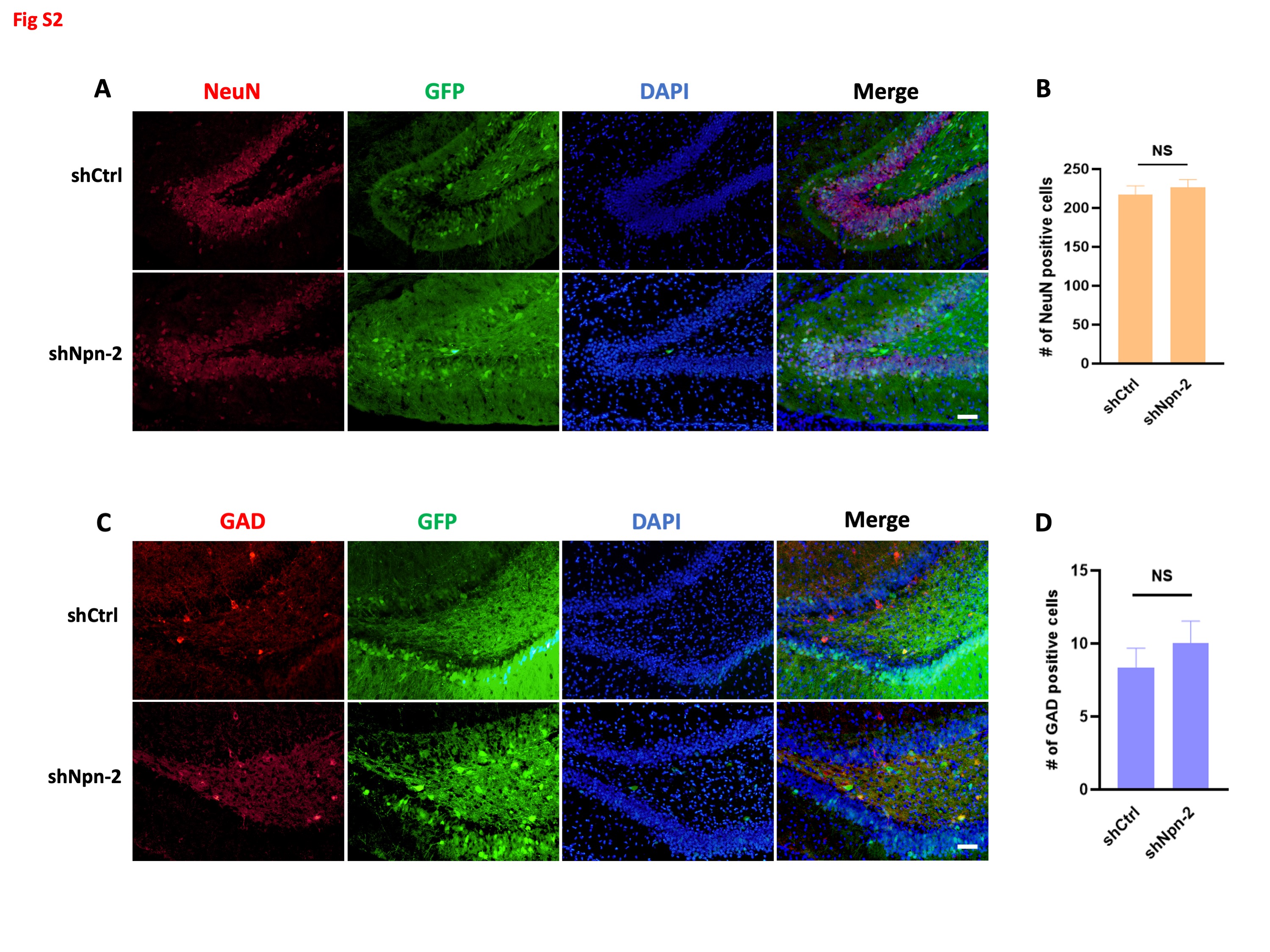


**Figure S2. Npn-2 knockdown in adult hippocampus had no effect on GABAergic interneurons.**

(A) No obvious neuron loss was found in Npn-2 knockdown animals. 14 days after AAV injection, rats in shCtrl group and shNpn-2 group were sacrificed for immunofluorescent staining with NeuN, a marker for neurons. No difference of neuron number in dentate gyrus was found between two groups. Scale bar, 100 μm.

(B) Quantitation of neuron number in A. n=3, P>0.05.

(C) Npn-2 knockdown had no effect on GABAergic interneurons. Representative pictures of brain slice immunofluorescent stained with GAD65/67, a marker for GABAergic interneurons. No difference of GAD positive cell number in dentate gyrus was observed between shCtrl group and shNpn-2 group at 14 days after intrahippocampal virus injection. Scale bar, 100 μm.

(D) Quantitation of the number of GABAergic interneurons in C. n=3, P>0.05.

Unpaired t-test. Error bars represent SEM.


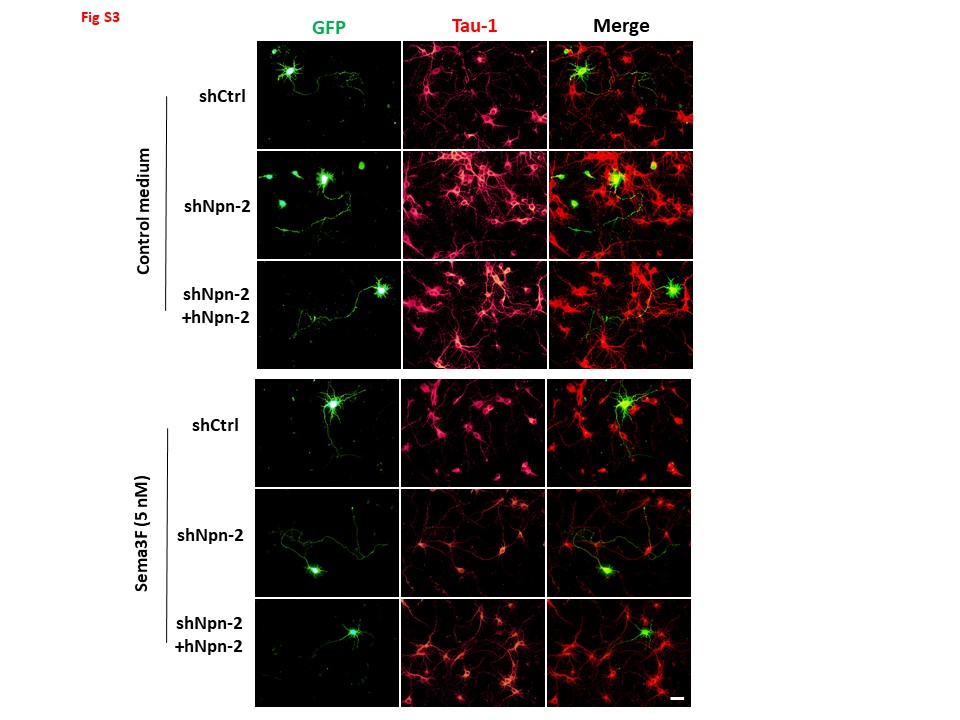


**Figure S3. Npn-2 signaling controls axon collateral formation.**

Primary hippocampal neurons were transfected with shCtrl, shNpn-2, or shNpn2 plus hNpn2 plasmids at DIV 0 and treated with control medium or 5 nM AP-sema3F at 48 hours after transfection for 24 hours. Representative immunofluorescent pictures of axons and their collaterals labeled with GFP (green) and Tau-1 (red), the marker of axon. This is the supplemental instruction for Figure 5. Scale bar, 40 μm.


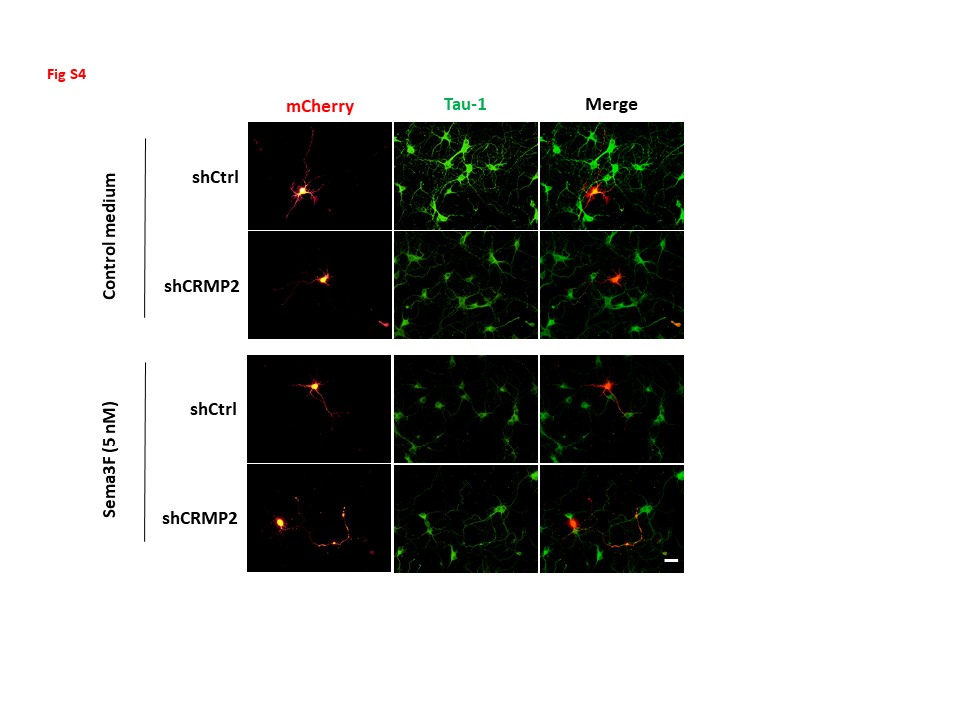


**Figure S3. Npn-2 signaling controls axon collateral formation.**

Primary hippocampal neurons were transfected with shCtrl, shNpn-2, or shNpn2 plus hNpn2 plasmids at DIV 0 and treated with control medium or 5 nM AP-sema3F at 48 hours after transfection for 24 hours. Representative immunofluorescent pictures of axons and their collaterals labeled with GFP (green) and Tau-1 (red), the marker of axon. This is the supplemental instruction for Figure 3. Scale bar, 40 μm.

**Figure S4. CRMP2 mediates Sema3F/Npn-2 signaling in axon collateral formation.**

Primary hippocampal neurons were transfected with shCtrl or shCRMP2 plasmid at DIV 0 and treated with control medium or 5 nM AP-sema3F at 48 hours after transfection for 24 hours. Representative immunofluorescent pictures of axons and their collaterals labeled with mCherry (red) and Tau-1 (green), the marker of axon. This is the supplemental instruction for Figure 7. Scale bar, 40 μm.
